# Dr. Horace Hoyt

**Published:** 1896-07

**Authors:** 


Dr. Horace Hoyt, of East Aurora, died at his home in that village, Wednesday, June 17, 1896, aged 73 years. Dr. Hoyt was a native of East Aurora, received his preliminary education at East Aurora Academy, and graduated in medicine at Buffalo University Medical College in 1847. He practised medicine in his native place more than forty years, having retired from active work about ten years ago. His father, Dr. Jonathan Hoyt, came to East Aurora, July 4, 1817. In 1851 Dr. Hoyt married Sarah Josephine Ballard, who died in October, 1886. Four children survive, one of whom is William B. Hoyt, a well-known attorney of Buffalo. The others are Albert H. Hoyt, Orson L. Hoyt, and Jennie D. Hoyt, of East Aurora. At the time of his death Dr. Hoyt was one of the trustees of the City and County Hall.

